# Therapeutic Effects of Topical Application of *Lycium barbarum* Polysaccharide in a Murine Model of Dry Eye

**DOI:** 10.3389/fmed.2022.827594

**Published:** 2022-03-11

**Authors:** Danyi Qin, Yingping Deng, Lixiang Wang, Hongbo Yin

**Affiliations:** Department of Ophthalmology, West China Hospital of Sichuan University, Chengdu, China

**Keywords:** *Lycium barbarum* polysaccharide, dry eye disease, inflammation, ocular surface, cornea

## Abstract

**Purpose:**

To evaluate the safety and efficacy of *Lycium barbarum* polysaccharide (LBP) eye drops in a murine model of dry eye disease (DED).

**Methods:**

Six- to eight-week-old female C57BL/6 mice were subjected to a combination of desiccating stress (DS) and topical benzalkonium chloride (BAC) to induce DED. Five microliters of LBP eye drops (0.625, 2.5, or 12.5 mg/ml) or PBS was applied topically 3 times per day for 10 days to subsequently test their efficacy. Tear secretion, tear breakup time (TBUT), corneal irregularity, and corneal fluorescein staining scores were measured on days 3 and 10 after treatment. The expression of tumor necrosis factor-alpha (TNF-α) in the cornea was assessed by quantitative (q) RT–PCR on days 10. The ocular irritation of LBP eye drops of corresponding concentrations was evaluated on 10- to 12-week-old female Sprague–Dawley rats.

**Results:**

Compared with PBS-treated groups, mice treated with 0.625, 2.5, and 12.5 mg/ml LBP showed a significant improvement in the clinical signs of DED in a dose-dependent manner, including corneal epithelial integrity, corneal regularity, and tear production, as well as significant inhibition of inflammatory cell infiltration and TNF-α expression levels in the cornea. All corresponding concentrations of LBP eye drops revealed no obvious ocular irritation.

**Conclusion:**

Topical application of LBP could ameliorate dry eye in a murine model of DED without obvious ocular irritation.

## Introduction

Dry eye disease (DED) is a chronic multifactorial ocular surface disease characterized by the loss of homeostasis of the tear film (1), affecting 5–50% of the population on a global scale (2). Ocular manifestations of DED include ocular discomfort, vision fluctuation, and potential damage to the ocular surface. The fundamental mechanism of DED is the vicious cycle caused by tear film hyperosmolarity and ocular surface inflammation, in which mitogen-activated protein kinase (MAPK) and nuclear factor kappa B (NF-κB) are the main signaling pathways (3). Glucocorticoids, non-steroidal anti-inflammatory drugs (NSAIDs), and immunosuppressive agents are the most popular topical anti-inflammatory agents for DED (4). However, local and systemic side effects are relatively common and restrict their application in some cases (5–7). Thus, safe and effective DED treatments are under active exploration.

Wolfberry (*Lycium barbarum* or goji berry) has been used as a valuable traditional Chinese medicine for more than 2,000 years, whose main active ingredient is Lycium barbarum polysaccharide (LBP) (8). Versatile functions of LBP, including immune-regulatory, anti-oxidative, anti-aging, and anti-inflammatory effects, have been revealed in several animal models of systemic diseases (9–11), as well as ocular fundus diseases (12–14). However, there is no relevant study about its anti-inflammatory effect on the ocular surface, especially with the drug delivery route of topical administration. Since inflammation plays a vital role in the progression of DED, LBP is likely to take effect.

In the current study, we investigated the safety and efficacy of LBP eye drops in a murine model of DED and explored their potential mechanisms to provide primary data for LBP ophthalmic preparations.

## Materials and Methods

### Preparation of *Lycium barbarum* Polysaccharide Eye Drops

*Lycium barbarum* polysaccharide (Catalog No. SP9311; Solarbio, Beijing, China) was diluted in sterile phosphate-buffered saline (PBS) to final concentrations of 0.625, 2.5, and 12.5 mg/ml. The eye drops were all freshly prepared and stored at 4°C prior to their use.

### Animals

Ten- to twelve-week-old female Sprague–Dawley (SD) rats and six- to eight-week-old female C57BL/6 (B6) mice were obtained from SPF (Beijing) Biotechnology Co., Ltd. All animals were kept under standard laboratory conditions with 12-h/12-h light-dark cycles, with food and water *ad libitum*. All animals were acclimated to the experimental environment for at least 1 week before the beginning of the study. Efforts were made to minimize the number of experimental animals and their suffering. All procedures conformed to the Association for Research in Vision and Ophthalmology Statement for the Use of Animals in Ophthalmic and Vision Research, and the present study was approved by the Animal Care and Use Committee (IACUC) of National Chengdu New Drug Safety Evaluation Center (approval number S2019022-P015-04).

### Mouse Model of Dry Eye

Desiccating stress (DS) was created by subcutaneous injection of 0.025 g/kg scopolamine hydrobromide (Chengdu YiRui Bio-Technology, Co., Ltd, Chengdu, batch number YRQ098-200901) 3 times daily (10 a.m., 1 p.m., and 4 p.m.) and exposure to a mini fan in a controlled dry environment (airflow rate of 15 L/min, relative humidity of 30–40%) 8 h per day for 7 days. This approach has been demonstrated to successfully induce the dry eye condition in mice (15). To enhance the ocular surface inflammation, 0.3% benzalkonium chloride (BAC) (Sigma–Aldrich, St. Louis, MO, United States, B6295) was topically applied on the ocular surface 3 times daily (10 a.m., 1 p.m., and 4 p.m.) simultaneously, as described by Zhang (16). A group of age-matched mice that did not receive any treatment served as non-stressed (NS) controls.

### Efficacy of *Lycium barbarum* Polysaccharide Eye Drops on B6 Mice

To evaluate the efficacy of LBP on the ocular surface of dry eye, 5 μl topical LBP was applied to B6 mice subjected to DS and BAC at low doses (LLBP, 0.625 mg/ml), medium doses (MLBP, 2.5 mg/ml), or high doses (HLBP, 12.5 mg/ml) 3 times daily (10 a.m., 1 p.m., and 4 p.m.) for 10 days. Animals in the vehicle control group and NS control group were given topical PBS with the same dosing regimen. The effects were evaluated with the tear secretion test, tear breakup time (TBUT), corneal fluorescein staining, and corneal irregularity test on days 3 and days 10 after treatment. Mice were then euthanized, and the globes were collected for histology study at the end of the study.

Tear production was measured with phenol red cotton threads (Tianjin Jingming Technological Development Co., Ltd., Tianjin, China). The thread was placed at the lateral 1/3 of the lower eyelid margin for 1 min, and the length of the wet part was measured.

To measure the TBUT, 2 μl of 0.1% sodium fluorescein was applied to the conjunctival sac. Mice were manually allowed to blink three times, and the time from the last blink to the first onset of breaking spots on the ocular surface was observed with the cobalt blue filter of a slit-lamp microscope (66 Vision Tech Co., Ltd., Hangzhou, China). In addition, corneal staining with fluorescein was observed and graded following the standard by the Chinese consensus of DED (17). Briefly, the cornea was equally divided into four quadrants and scored individually: Absent, 0; punctate staining ≤ 30 spots, 1; punctate staining > 30 spots but not diffuse, 2; diffuse staining, 3. The scores of each quadrant were summed (maximum, 12 points).

Corneal irregularity scores were evaluated by reflecting a ring light on the ocular surface to assess corneal smoothness (18). Corneal irregularity was scored as follows: no distortion, 0; distortion in one quadrant, 1; distortion in two quadrants, 2; distortion in three quadrants, 3; distortion in all four quadrants, 4; severe distortion and no ring shape recognizable, 5.

### Safety Evaluation

To evaluate the safety of LBP eye drops on the ocular surface, LBP treatment was performed on SD rats by topical application of 10 μl LBP (0.625, 2.5, or 12.5 mg/ml) or vehicle (PBS) eye drops 3 times daily (10 a.m., 1 p.m., and 4 p.m.) for 7 days. Blank eyes with no treatment were used as a non-treatment control. Ocular irritation was observed on days 1, 2, 3, and 7 after treatment. Irritation of the cornea, iris, and conjunctiva was separately scored on a 0–4 scale and summed to a total grade following the standard of the Draize scale scoring system (maximum 100 points) (19).

### Histology Study

Both the eyes and adnexa of SD rats and the right eyes and adnexa of B6 mice from each group were surgically excised after euthanasia at the end of the study. The tissues were fixed in 10% formalin solution, trimmed, embedded in paraffin, cut into serial sagittal sections (5 μm thick), and stained with hematoxylin and eosin (H&E) following standard protocols. The slides were imaged using a DP72 digital camera (Olympus, Tokyo, Japan). The cell types were identified by the morphology. The density of inflammatory cells (polymorphonuclear and monocytes) in the cornea was manually counted in 10 cross-sectional high-power fields (HPFs) at 40× magnification that evenly distribute from the nasal to the temporal limbus.

### Corneal TNF-α Level

The relative mRNA expression level of TNF-α in the cornea of the left eyes of B6 mice was examined with quantitative reverse-transcription polymerase chain reaction (qRT–PCR). The cornea was carefully excised from the globe, immediately frozen with liquid nitrogen, and transferred to a -70^°^C refrigerator for storage before the test. Total RNA was extracted using a total RNA isolation kit (Catalog no. R1200; Solarbio, Beijing, China). First-strand cDNA was synthesized using a reverse transcription kit (Catalog no. FP205; TIANGEN, Beijing, China). Real-time PCR was performed on a real-time PCR system (Q2000A Biosystems, LongGene, Hangzhou, China). The primers are provided in Table 1. The parameters were predenaturation at 95°C for 15 min, followed by 40 cycles of denaturation at 95°C for 10 s, annealing for 20 s, and final extension at 72°C for 30 s. A melting curve analysis was conducted to check the amplification specificity. Samples and standards were assessed in duplicate. The 2^–ΔΔCt^ method was employed to calculate the relative TNF-α expression levels using β-actin as a reference gene (20, 21).

**TABLE 1:** The primers sequence.

Gene	Primer	The primers sequence
TNF-α	Forward	5′-CTGAACTTCGGGGTGATCGG-3′
	Reverse	5′-GGCTTGTCACTCGAATTTTGAGA-3′
β-actin	Forward	5′-GTCCCTCACCCTCCCAAAAG-3′
	Reverse	5′-GCTGCCTCAACACCTCAACCC-3′

### Statistical Analysis

Continuous data following a normal distribution are represented as the mean ± standard deviation (SD); otherwise, they are represented as the median (interquartile range) (IQR). Statistical significance was evaluated by multiway ANOVA with Tukey’s *post-hoc* test or by Kruskal–Wallis test when appropriate using SPSS Statistics 26.0 (IBM, Armonk, NY, United States) and GraphPad Prism 5.0 software (GraphPad Software; San Diego, CA, United States). *P* < 0.05 was considered statistically significant.

## Results

### Topical Application of *Lycium barbarum* Polysaccharide Improved Corneal Epithelial Integrity and Regularity

**FIGURE 1:**
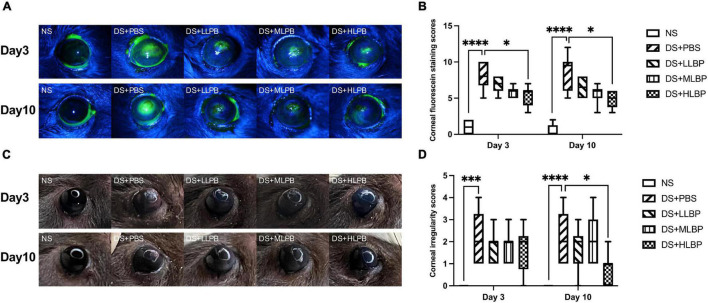
The effects of topical application of LBP on corneal epithelial integrity and regularity in a mouse DED model. **(A)** Representative images of corneal fluorescein staining in different groups on days 3 and 10 after treatment. **(B)** The results of corneal fluorescein staining scores of different groups on days 3 and 10 after treatment. **(C)** Representative images of corneal irregularity in different groups on days 3 and 10 after treatment. **(D)** The comprehensive results of corneal irregularity scores of different groups on days 3 and 10 after treatment. Data were shown as median (IQR). *P < 0.05, ^***^P < 0.001, ^****^P < 0.0001.

The corneal fluorescein staining scores significantly increased in the DS + PBS group compared to the NS group 7 days after modeling [Figure 1B; NS vs. DS + PBS, 1.5 (2.25) vs. 12 (0.75), *P* < 0.0001]. As shown in Figures 1A,B, compared with PBS treatment, topical application of HLBP significantly decreased corneal fluorescein staining on both days 3 [Figure 1B; DS + PBS vs. DS + HLBP, 8 (3.25) vs. 6 (2); *P* < 0.05] and days 10 [Figure 1B; DS + PBS vs. DS + HLBP, 9.5 (4) vs. 5 (2.25); *P* < 0.05]. The corneal irregularity scores were significantly increased in the DS + PBS group compared with the NS group [Figure 1D; NS vs. DS + PBS, 0 (0) vs. 2 (2), *P* < 0.0001]. As shown in Figures 1C,D, topical application of HLBP significantly improved corneal surface regularity compared with the DS + PBS group on days 10 [Figure 1D; DS + PBS vs. DS + HLBP, 2 (2.25) vs. 1 (1); *P* < 0.05].

### Topical Application of *Lycium barbarum* Polysaccharide Improved the Tear Secretion Rate and Tear Film Stability

**FIGURE 2:**
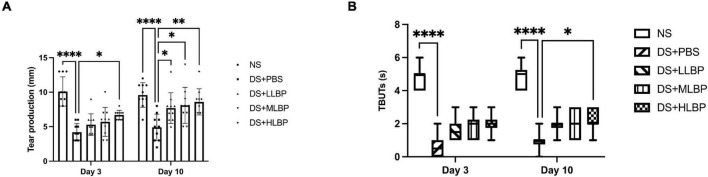
**(A)** The effects of topical application of LBP on the tear secretion rate on days 3 and 10 after treatment. **(B)** The effects of topical application of LBP on TBUTs on days 3 and 10 after treatment. Data of tear secretion rate were shown as the mean ± SD. Data of TBUTs were shown as median (IQR). *P < 0.05, ^**^P < 0.01, ^****^P < 0.0001.

Compared to the NS group, the DS + PBS group showed a significantly decreased tear secretion rate (Figure 2A; NS vs. DS + PBS, 10.400 ± 2.836 mm/min vs. 1.825 ± 1.107 mm/min, *P* < 0.0001). Topical application of HLBP significantly increased the tear secretion rate on days 3 (Figure 2A; DS + PBS vs. DS + HLBP, 4.200 ± 1.229 mm vs. 6.700 ± 0.6749 mm; *P* < 0.05), and topical application of LLBP, MLBP, and HLBP all significantly increased the tear secretion rate on days 10 (Figure 2A; DS + PBS vs. DS + LLBP, 4.900 ± 1.912 mm vs. 7.700 ± 2.214 mm; *P* < 0.05; DS + PBS vs. DS + MLBP, 4.900 ± 1.912 mm vs. 8.100 ± 2.601 mm; *P* < 0.05; DS + PBS vs. DS + HLBP, 4.900 ± 1.912 mm vs. 8.600 ± 1.897 mm; *P* < 0.01). These results suggested that topical application of LBP could improve tear production during DS.

TBUTs significantly decreased in the DS + PBS group compared with the NS group [Figure 2B; NS vs. DS + PBS, 4.5 (2.25) s vs. 0 (0) s, *P* < 0.0001]. After 10 days of topical treatment with HLBP, the TBUTs significantly improved compared with PBS treatment [Figure 2B; DS + PBS vs. DS + HLBP, 1 (0.25) s vs. 2 (1) s; *P* < 0.05].

### Topical Application of *Lycium barbarum* Polysaccharide Inhibited Ocular Surface Inflammation

**FIGURE 3:**
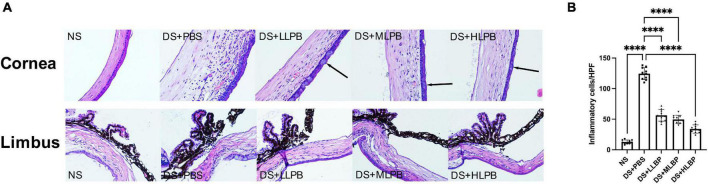
**(A)** Representative images of H&E staining in the cornea and limbus of different groups on days 10 after treatment. The arrows showed corneal conjunctivization (magnification: 40×). **(B)** The inflammatory cell count in the cornea and limbus of different groups on days 10 after treatment. ^****^P < 0.0001.

Compared with the NS group, the DS + PBS group showed obvious inflammatory cell infiltration on the ocular surface (Figure 3A; NS vs. DS + PBS, 12.700 ± 3.199/HPF vs. 124.400 ± 9.743/HPF, *P* < 0.0001), accompanied by corneal edema and neovascularization. In addition, corneal conjunctivization could be observed in some cases in the DS groups. Topical application of LBP effectively suppressed the DS-induced infiltration of inflammatory cells on the ocular surface in a dose-dependent manner (Figure 3B; DS + PBS vs. DS + LLBP, 124.400 ± 9.743/HPF vs. 56.300 ± 9.581/HPF; *P* < 0.0001; DS + PBS vs. DS + MLBP, 124.400 ± 9.743/HPF vs. 49.600 ± 6.637/HPF; *P* < 0.0001; DS + PBS vs. DS + HLBP, 124.400 ± 9.743/HPF vs. 33.900 ± 6.839/HPF; *P* < 0.0001). Edema and neovascularization in the cornea, limbus, and iris were also alleviated after treatment.

### Topical Application of *Lycium barbarum* Polysaccharide Reduced TNF-α Production in the Cornea of B6 Mice

**FIGURE 4:**
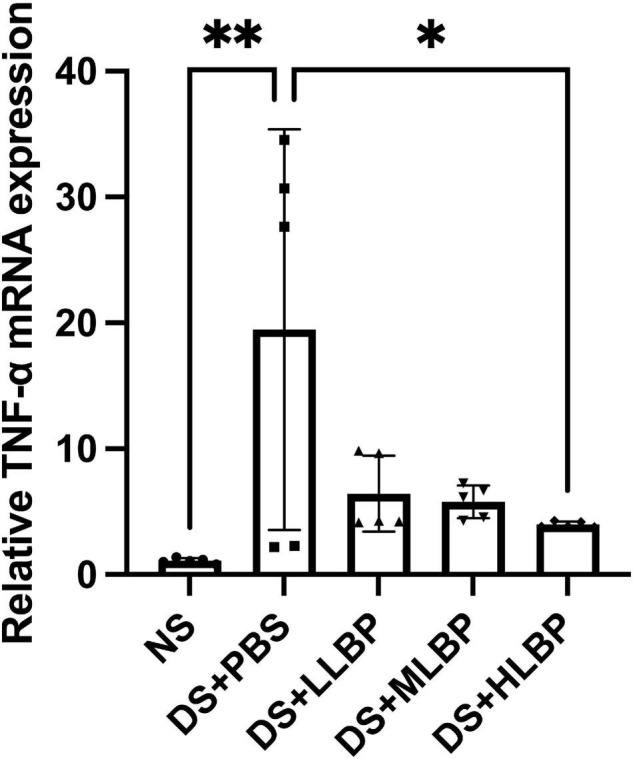
The effects of topical application of LBP on the relative expression level of TNF-α mRNA in the cornea. Data were shown as the mean ± SD. *P < 0.05, ^**^P < 0.01.

The relative expression level of TNF-α mRNA in the cornea significantly increased in the DS + PBS group compared to the NS group (Figure 4; NS vs. DS + PBS, 1.100 ± 0.213 vs. 19.470 ± 15.920; *P* < 0.01). Topical application of HLBP for 10 days significantly decreased the relative TNF-α expression level in the cornea (Figure 4; DS + PBS vs. DS + HLBP, 19.470 ± 15.920 vs. 3.989 ± 0.236; *P* < 0.05).

### Topical Application of *Lycium barbarum* Polysaccharide Did Not Induce Irritations or Pathological Changes on the Ocular Surface in Sprague–Dawley Rats

**FIGURE 5:**
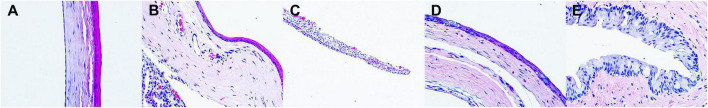
Representative images of H&E staining of ocular tissues on days 7. **(A)** the cornea; **(B)** the limbus; **(C)** the iris; **(D)** the bulbar conjunctiva; **(E)** the fornix conjunctiva (magnification: 40×).

Tables 2, 3 demonstrate the results of the safety test evaluated by the Draize scale scoring system and corneal fluorescein staining, respectively. For all time points and tissues, the scores of each group remained nearly constant. There were no significant differences in the Draize or fluorescence scores among all groups at any time point. Figure 5 shows the results of H&E staining of the ocular surface from the HLBP group on days 7. There was no congestion or edema in the conjunctiva. The structure of the iris and each layer of the cornea remained normal. No obvious inflammatory cell infiltration or other histopathological changes were observed.

**TABLE 2:** The comprehensive results of ocular surface irritation scored following the Draize scale scoring system.

Draize scores [median (IQR)]	NT	PBS	LLBP	MLBP	HLBP	*P*-value
Day 0	0 (0)	0 (0)	0 (0)	0 (0)	0 (0)	1.000
Day 1	0 (0)	1 (2)	1 (2)	3 (2)	0 (0)	0.213
Day 2	0 (0)	0 (0)	1 (2)	1 (2)	0 (0)	0.497
Day 3	0 (0)	0 (0)	0 (0)	2 (2)	0 (0)	0.061
Day 7	0 (0)	0 (0)	0 (0)	1 (2)	0 (0)	0.406

**TABLE 3:** The results of corneal fluorescein staining scores in the safety test.

Corneal fluorescein scores [median (IQR)]	NT	PBS	LLBP	MLBP	HLBP	*P*-value
Day 0	0 (0)	0 (2)	0 (0)	0 (0)	0 (0)	0.406
Day 1	0 (0)	0 (0)	0 (0)	0 (0)	0 (0)	1.000
Day 2	0 (0)	0 (0)	0 (0)	0 (0)	0 (0)	1.000
Day 3	0 (0)	0 (0)	0 (0)	0 (0)	0 (0)	1.000
Day 7	0 (0)	0 (0)	0 (0)	0 (0)	0 (0)	1.000

## Discussion

Dry eye disease is one of the most common chronic inflammatory ocular surface diseases with an increasingly high prevalence. Safe and effective anti-inflammatory therapies are always needed. LBP, a water-soluble polysaccharide, possesses an effect of anti-inflammation. However, very few studies concentrate on its effects on ocular surface diseases. Moreover, the methods of LBP administration in almost all the studies are oral administration or gavage, rather than topical application *via* eye drops. Therefore, this study evaluated the ocular surface irritation and the effects of LBP eye drops on DED.

Our findings demonstrated that topical application of LBP could effectively improve corneal epithelial integrity, corneal regularity, and tear production in a murine DED model induced by DS and BAC. It could also decrease inflammatory cell infiltration, alleviate edema of the ocular tissues, and reduce the expression of TNF-α in the cornea. It showed little irritation or toxicity to the ocular surface. These results are consistent with our hypothesis.

Subcutaneous injection of scopolamine hydrobromide combined with DES is commonly used to establish DED models in mice (15). However, the dry eye symptoms are self-limited and begin to subside once DS is eliminated in our previous work, which is consistent with Yoon’s study (22). Topical administration of BAC drops is another valid approach to induce dry eye (16). Therefore, we combined DS and BAC to acquire a stable and reliable murine DED model, whose severe manifestations of dry eye persisted through the end of our study and could be ameliorated by topical treatment with LBP.

Ocular surface inflammation induced by tear film instability and tear hyperosmolarity plays a vital role in the pathogenesis and progression of chronic DED (1). Inflammatory cytokines, such as interferon-1 (IL-1) produced mainly by Th1 cells and TNF-α produced mainly by Th17 cells, are elevated in the cornea and conjunctiva of dry eye (3). TNF-α is significantly increased in the tears of dry eye patients as one of the most common pro-inflammatory cytokines. TNF-α triggers several signal transduction pathways, such as NF-κB, p38 MAPK, extracellular signal-regulated kinase (ERK), and c-Jun N-terminal kinase (JNK), by interacting with TNF receptor 1 (TNFR1) and TNF receptor 2 (TNFR2) on the ocular surface (23, 24). TNF-α is considered to be correlated with disease severity, including OSDI score and tear secretion. It can stimulate matrix metalloproteinase (MMP) activation, promote mononuclear cell infiltration into glands, and enhance the apoptosis of glandular epithelial cells (25, 26). In addition, numerous attempts have been made to develop TNF-α blockers as potential therapeutic agents for the treatment of inflammatory diseases (27). Thus, TNF-α could serve as an inflammatory biomarker of DED. Meanwhile, the mechanism of the anti-inflammatory effects of LBP in many systemic diseases was found to be related to TNF-α and its upstream and downstream pathways (9, 10, 28, 29). In our study, the expression of TNF-α in the cornea significantly increased in the murine DED model, and decreased after the treatment of HLBP. Accordingly, we speculated that LBP eye drops inhibited the inflammation on the ocular surface by decreasing TNF-α, which needs to be further evaluated in the future studies.

This is the first study to investigate the safety and efficacy of LBP eye drops in treating DED. Our study uncovered that topical application of LBP alleviated dry eye symptoms and inhibited ocular surface inflammation in a murine DED model, providing the primary data for LBP ophthalmic preparations. Additionally, LBP has an advantage over other anti-inflammatory drugs due to its easy availability in natural plants and low price, without toxicity or short-term side effects. However, there are several limitations in our study. The underlying mechanism is still unclear. Further studies are needed to explore the specific anti-inflammatory pathways of LBP in DED treatment. The long-term efficacy and safety remain to be determined.

## Data Availability Statement

The original contributions presented in the study are included in the article/Supplementary Material, further inquiries can be directed to the corresponding author.

## Ethics Statement

The animal study was reviewed and approved by the Animal Care and Use Committee (IACUC) of National Chengdu New Drug Safety Evaluation Center (approval number S2019022-P015-04).

## Author Contributions

DQ, YD, LW, and HY performed the material preparation and data collection and analysis. DQ wrote the first draft of the manuscript. All authors contributed to the study conception and design, commented on previous versions of the manuscript, and read and approved the final manuscript.

## Conflict of Interest

The authors declare that the research was conducted in the absence of any commercial or financial relationships that could be construed as a potential conflict of interest.

## Publisher’s Note

All claims expressed in this article are solely those of the authors and do not necessarily represent those of their affiliated organizations, or those of the publisher, the editors and the reviewers. Any product that may be evaluated in this article, or claim that may be made by its manufacturer, is not guaranteed or endorsed by the publisher.
